# Targeted Tick-Borne Disease Recognition: Assessing Risk for Improved Public Health

**DOI:** 10.3390/healthcare12100984

**Published:** 2024-05-10

**Authors:** Pyung Kim, Sarah Maxwell, Nabila Parijat, Dohyeong Kim, Connie L. McNeely

**Affiliations:** 1 School of Economic, Political and Policy Sciences, University of Texas at Dallas, Richardson, TX 75080, USA; pyung.kim@utdallas.edu (P.K.); nabila.parijat@utdallas.edu (N.P.); dohyeong.kim@utdallas.edu (D.K.); 2 Schar School of Policy and Government, George Mason University, Fairfax, VA 22030, USA

**Keywords:** tick-borne disease, prevention, risk assessment, Lyme disease, vulnerable populations, occupational risk, pet ownership

## Abstract

Tick-borne diseases (TBDs) pose a rapidly growing threat to public health. The incidence of TBDs is on the rise, necessitating a comprehensive understanding of the risk factors beyond demographic considerations. This brief report combines a preliminary review of the literature with geographical case mapping to identify the various factors influencing TBD risk. The report highlights the vulnerability of outdoor workers, the importance of outdoor activities, and the role of education in adopting preventive behaviors. Pet ownership and interactions with animals are also associated with an increased risk. The state of Illinois is used as a case study for this report, revealing regional variations in TBD incidence, and linking them to agricultural practices, forested areas, and park accessibility. These findings inform recommendations for targeted prevention strategies, emphasizing the need for detailed geographical data to enhance public health efforts in curbing TBD incidence and risk.

## 1. Introduction

In the United States (U.S.), the four primary tick vectors of disease are *Ixodes scapularis*, *Amblyomma americanum*, *Dermacentor variabilis*, and *Ixodes pacificus* [1]. These ticks transmit a variety of tick-borne diseases (TBDs) to humans through three types of tick-borne pathogens: viruses, bacteria, and protozoa. For example, the vector *Ixodes scapularis* can transmit human granulocytic anaplasmosis, Lyme disease (LD), Relapsing fever, babesiosis, and Powassan encephalitis through pathogens such as *Anaplasma phagocytophilum*, *Borrelia burgdorferi*, *Borrelia miyamotoi*, *Babesia microti*, or *Powassan virus*. *Amblyomma americanum* can transmit human monocytic ehrlichiosis, Heartland virus disease, and Bourbon virus disease through *Ehrlichia chaffeensis*, Heartland virus, or Bourbon virus. *Dermacentor variabilis* is known to transmit Rocky Mountain spotted fever via *Rickettsia rickettsia*. *Ixodes pacificus* can transmit LD through *Borrelia burgdorferi* [1].

Tick-borne diseases (TBDs) have emerged as one of the most rapidly growing threats to public health in the U.S. today and in other countries. For instance, the number of annually reported LD cases, a significant subset of TBDs, in the U.S. has more than doubled since 2000, potentially exceeding 476,000 [2]. Alongside LD, other vectors and pathogens also have been reported in various areas, including the Asian longhorned tick (*Haemaphysalis longicornis*), the American dog tick (*Dermacentor variabilis*), the lone star tick (*Amblyomma americanum*), the Gulf Coast tick (*Amblyomma maculatum*), and the black-legged tick, leading to growing cases of ehrlichiosis, anaplasmosis, and spotted fever rickettsioses, for example [3,4,5].

The incidence of TBDs is escalating nationwide, surpassing traditionally limited geographical strongholds in the Northeastern regions [5]. Additionally, there have been reports of persistent TBD infections in patients who continue to experience symptoms even after antibiotic treatment [6]. To address related problems, recent research has utilized various methods harnessing, for example, canine proxy data and analyzing patient self-reports, alongside other approaches, to identify county-level and other spatially delineated indicators of LD and the spread of other TBDs [7,8].

Nevertheless, the actual burden of TBDs in the U.S. is largely unknown. Public health systems and healthcare providers often lack comprehensive information regarding tick populations, pathogens, and the overall disease burden, especially in regions beyond the Northeast. Note that conventional approaches to assessing the risk factors associated with TBD infections often emphasize demographic factors. For instance, according to the Centers for Disease Control and Prevention (CDC), boys aged 5–9 and older males aged 65–79 are the most vulnerable groups [9]. When considering racial groups, it becomes evident that the majority of confirmed or probable LD cases are found among white individuals, with the “other” category comprising the largest proportion of the remaining cases, followed by Black and Asian individuals [9]. However, such approaches typically are not well contextualized and do little to delineate actual risk factors. Solely relying on this approach can be limiting when it comes to developing targeted prevention strategies for TBDs and does not provide specific behavioral guidelines. Furthermore, although medically important ticks have expanded into geographic areas previously considered non-endemic, resulting in increased human TBD cases [10], national maps that depict the distribution of medically significant ticks and the presence or prevalence of tick-borne pathogens are frequently incomplete, outdated, or absent [11].

We purport that the lack of knowledge related to the behaviors, activities, living conditions, and other factors associated with TBDs hinders prevention and diagnostic opportunities. This report takes a step in addressing such issues, identifying examples of demographic-related or delineated activities that may be linked to enhanced tick bite exposure and TBD risks. Specifically, we explore the risk factors associated with occupations, working conditions, activities, pet ownership, and animal encounters. In addition, we provide a geographical case study, employing thematic mapping to examine the differences in risk at local levels. Through this report, we offer a more varied approach to assessing TBD risk areas and address the lingering difficulties facing public health officials in relation to recognizing audiences with risk exposure.

## 2. Materials and Methods

Aimed at informing more precise and targeted TBD risk prevention strategies, we employed a primarily observational, short study that included a brief review of the related scholarly literature, drawing from conventional sources such as PubMed and Google Scholar, in addition to analytic thematic mapping techniques, to present visual representations of TBD risk. We scrutinized articles to extract insights on the risk factors associated with occupations, activities, the ownership of pets and livestock, and encounters with wildlife, as these were suggested as critical considerations in other research exploring TBD risk factors (e.g., [7,8]). Additionally, we examined studies that shed light on factors conducive to promoting preventive behaviors.

To evaluate the practical applicability of previous study findings regarding the risk factors for TBD infections, we employed a brief mapping approach, utilizing TBD data sourced from the Illinois Department of Public Health (IDPH). Illinois was selected for a more focused case study since the IDPH offers more detailed data on TBD incidents disaggregated at different levels of analysis—in this case, county-level—compared to the majority of other U.S. states and compared to the CDC, which principally releases only Lyme disease (LD) data in any detail. Specifically, the IDPH offers data on four types of TBD average incidence rates per 100,000 individuals annually, spanning from 2012 to 2021. These encompass the most commonly recognized TBDs, including LD, anaplasmosis, ehrlichiosis, and Spotted Fever Group Rickettsiosis (SFR). We utilized 10-year average TBD data since separate yearly data were not available. Leveraging this dataset, we created visual representations depicting the geographic distribution of TBDs at the county level.

To look into the relationship between human TBDs in Illinois and various contributing factors, such as those related to tick habitats, we collated additional county-level data, encompassing the percentage of land dedicated to pasture or hay in the year 2021, the extent of forest coverage in 2021, and the proportion of individuals residing within a one-mile radius of a park in 2020. Additionally, although boys and older males sometimes appear to be the most vulnerable [9], we collected data on the percentage of the adult population aged 25 and above holding a high school diploma or higher qualification in 2021, since previous studies have indicated positive correlations between higher education and knowledge about preventative behaviors for TBD infection [12]. Community characteristics data were sourced from the CDC. Armed with this county-level dataset, we conducted various mapping analyses using Tableau Desktop (version 2022.2).

## 3. Literature Review, Observations, and Thematic Mapping Case Study

### 3.1. Literature Review

A targeted brief review was used to identify the basic risk factors drawn from findings in the medical and public health scholarly literature. The results highlight examples of the localized or distinguishing factors associated with an increased risk of tick exposure or TBDs.

#### 3.1.1. Occupations Associated with TBDs

Occupations involving outdoor work have consistently shown a higher susceptibility to TBDs in various studies [13,14,15,16,17,18,19,20,21]. Among these, agricultural workers have been identified as a group that is particularly vulnerable to TBDs [13,17,19,22,23]. Similarly, forestry workers also exhibit a significantly elevated risk of TBDs [13,16,17,18,19,20,21,22,23], and occupational cavers are not exempt from this heightened risk [24].

However, it is essential to emphasize that the mere membership of these occupational categories does not inherently define risk level. Rather, the actual risk is influenced by the kinds of tasks performed and the working environment [13]. For example, although employees within the national park services generally face a relatively higher risk compared to other outdoor occupations, rangers and rescue crew members are at a relatively higher risk of tick bite exposure than those primarily involved in administrative or managerial roles among park service workers [14]. Similarly, among migrant and seasonal laborers in Texas, those who exhibited the most severe symptoms indicative of a TBD were more likely to have spent nights outdoors [25]. This underscores that the actual risk of TBDs is not solely determined by occupational categories, but that it also encompasses factors such as the duration of outdoor exposure.

#### 3.1.2. Activities Associated with TBDs

Numerous studies have consistently underscored a clear correlation between spending more hours engaged in outdoor activities each week and an increased risk of encountering ticks [26,27,28]. For instance, documented cases of TBD infections among children, campers, and staff following camp activities highlight this risk [29,30,31]. Similarly, scouts face an elevated risk of tick bites and TBD infections [32].

Engaging in daily outdoor recreational activities, such as visiting parks or hiking, has also been linked to testing positive for TBDs [33,34,35]. Furthermore, researchers have demonstrated that seemingly innocuous activities like walking on wooded pathways [36] or gardening [35] can lead to encounters with ticks. Consequently, it is advisable to take adequate precautions to ensure that ticks found on clothing are effectively removed through appropriate washing and drying procedures following outdoor activities [26,37,38].

#### 3.1.3. Educational Level, Knowledge about TBDs, and Preventative Behaviors

Having knowledge about ticks significantly predicts whether individuals take measures to prevent TBDs after spending time outdoors [12,18,39]. For instance, those with a bachelor’s degree or higher education are notably more likely to provide accurate answers related to ticks compared to individuals with a high school education or lower [12]. Additionally, individuals who have personally recovered from or have witnessed a family member recover from a tick-borne disease demonstrate a significantly higher level of correct answers than those without such personal experiences [12]. Conversely, individuals who do not adopt preventive measures often mention that some preventive strategies hinder their enjoyment of nature and express skepticism about their effectiveness [12]. They also believe it is unlikely that they would be bitten by ticks, yet simultaneously lack knowledge about what a tick bite looks like and how to properly remove a tick [26,40,41].

#### 3.1.4. Pet Ownership and Animal Encounters

Pet ownership and contact with animals have been identified as potential contributing factors to TBD infections [18,42,43,44,45,46,47,48]. The presence of pets, whether they live inside or outside the house, has shown a positive correlation with the risk of encountering ticks, as pets that venture outdoors may carry ticks indoors [43]. Specifically, research indicates that both dog owners and cat owners face an elevated risk of TBD exposure [18].

The risk of TBD infection extends beyond pets to other animals that can serve as sources of transmission. A comprehensive meta-analysis involving data from 69 global studies underscored a significantly heightened risk of TBDs among livestock owners and veterinarians [20], highlighting that contact with animals can substantially increase the risk of TBD transmission. Wildlife animals such as passerine birds [34,44], deer [45], wild pigs [46], and hedgehogs [47,48] also play a significant role as hosts for ticks, further emphasizing the potential for TBD transmission through various animal encounters.

### 3.2. Case Study on Illinois County-Level Proxy Variables for TBD Risk

#### 3.2.1. Distributions of TBDs in Illinois

Studies consistently indicate that working outdoors, engaging in outdoor activities, and residing in environments where individuals frequently encounter animals are associated with an increased risk of TBD infections. Moreover, having knowledge about ticks is more likely to lead to the adoption of preventive behaviors. To assess the practical relevance of these findings from previous research on TBD infection risk factors, we conducted a case mapping analysis using TBD data from Illinois.

**Figure 1:**
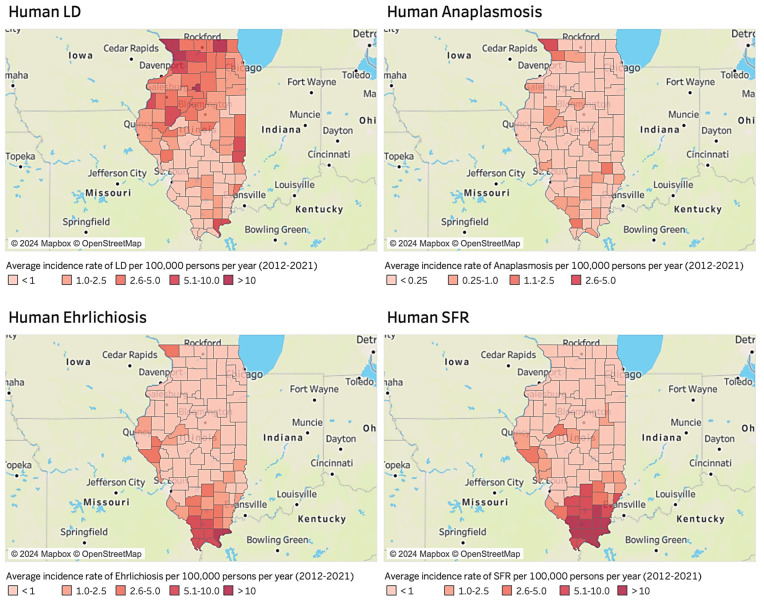
Average incidence rate of human TBDs per 100,000 persons per year in Illinois (2012–2021).

First, Figure 1 illustrates the distribution of the average incidence rates for human LD, anaplasmosis, ehrlichiosis, and SFR per 100,000 persons per year between 2012 and 2021 (Figure 1). The average rates of human LD and anaplasmosis are high in the northwestern region, while those of human ehrlichiosis and SFR are elevated in the southern region, which is relatively distant from Chicago and Saint Louis. To gain further insights, we compared these distributions of human TBDs with mapping data related to factors such as land used for agriculture, land covered by forest, park accessibility, and educational level.

#### 3.2.2. Proxy Variables for TBD Risk

**Figure 2:**
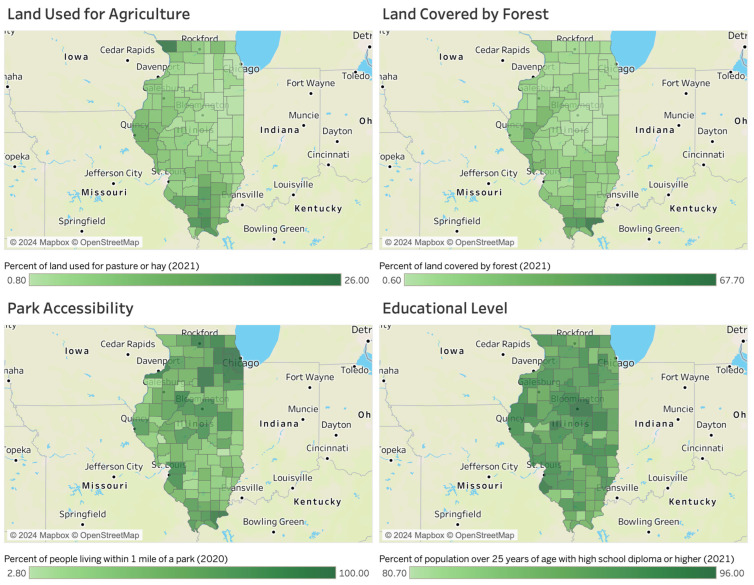
Land used for agriculture, land covered by forest, park accessibility, and educational levels in Illinois.

Figure 2 presents a visual representation of various factors in Illinois, including agricultural land use (upper left), forested land cover (upper right), park accessibility (lower left), and educational levels (lower right). First, the percentage of land used for agriculture is notably high in the northwestern and southern regions of Illinois, coinciding with areas where the human TBD incidence rates are elevated. This aligns with prior research that identified agricultural workers as having a relatively higher risk of TBD infection.

Secondly, the percentage of forest land coverage is notably higher in the southern region, where there is a greater prevalence of human ehrlichiosis and SFR cases. Additionally, this densely forested region may also witness increased levels of outdoor activity and a higher likelihood of encounters with animals. This observation aligns with prior research that identified an elevated risk of Tick-Borne Disease (TBD) infection among forestry workers.

Third, park accessibility, measured as the percentage of people living within 1 mile of a park, is notably high in the northeastern, central, and southern regions of Illinois. This may be related to the likelihood of participating in outdoor activities, as previous research has identified such activities as potential risk factors. However, it is worth noting that while the southern region has a higher ehrlichiosis and SFR infection rate, this pattern does not necessarily hold true for other regions.

Lastly, some previous literature has found that individuals with higher levels of education are more likely to engage in behaviors aimed at preventing TBDs [12,22,39]. However, establishing a clear geographical relationship between educational levels and TBD infection rates may prove more complex.

## 4. Conclusions and Observations

This report combined a brief review of the risk factors associated with various elements, including occupations, working conditions, activities, pet ownership, and encounters with animals, with a geographical case study employing thematic mapping to scrutinize regional variations in risk using Illinois data. These observations could inform recommendations to effectively guide the identification of at-risk audiences, behaviors, messages, communication platforms, and geographical information in the prevention of TBDs.

For instance, recommended strategies encompass the application of tick pesticides in wooded areas, the adoption of routine tick checks following outdoor activities, the installation of tall fences to deter deer from entering properties, and the trimming of shrub edges to minimize TBD risk. It is also prudent to prioritize public health communication campaigns that disseminate messages promoting preventive behaviors in areas with elevated risk profiles. These high-risk regions may be characterized by significant agricultural land, densely forested areas, or increased accessibility to parks.

Geographical information, which can enhance the effectiveness of these guidelines, is often overlooked and conspicuously absent in the existing literature. Furthermore, the CDC seldom releases data for TBDs, except for LD, at scales other than at national or state levels, which limits the capacity to tailor prevention strategies with precision. Consequently, public health policy often takes a broader, less specific approach to prevention efforts. These insights hold significant relevance for informing the public health experts and decision-makers involved in crafting strategic communication campaigns to combat TBDs. For greater precision, the necessity of supplying more comprehensive and finely segmented TBD data, surpassing the national or state level, is evident in the formulation of practical and targeted guidelines.

In addressing this need, we emphasize the critical role of local health departments and community organizations in gathering and disseminating TBD data. By leveraging local knowledge and resources, these entities can fill the existing data absence, providing a more accurate and actionable picture of TBD risks. Additionally, the integration of technology, such as geographic information systems (GISs) and citizen science platforms, can enhance data collection efforts, offering real-time insights into the evolving landscape of TBD risks.

Furthermore, the findings highlight the importance of intersectoral collaboration between public health authorities, environmental agencies, and the community at large. Such partnerships are essential in developing comprehensive strategies that address the complex interplay of factors influencing TBD risk. From enhancing landscape management to fostering community engagement in prevention efforts, a collaborative approach can significantly amplify the impact of TBD mitigation strategies.

In conclusion, this report lays the groundwork for a more targeted and effective approach to TBD prevention, calling for enhanced geospatial data collection efforts, innovative public health messaging, and strengthened intersectoral collaboration. As we move forward, the insights gleaned from this report will be instrumental in guiding the development of multifaceted public health strategies that are responsive to the specific needs of at-risk communities.

## Data Availability

Data are available upon request.
